# Machine Learning Algorithms to Detect Subclinical Keratoconus: Systematic Review

**DOI:** 10.2196/27363

**Published:** 2021-12-13

**Authors:** Howard Maile, Ji-Peng Olivia Li, Daniel Gore, Marcello Leucci, Padraig Mulholland, Scott Hau, Anita Szabo, Ismail Moghul, Konstantinos Balaskas, Kaoru Fujinami, Pirro Hysi, Alice Davidson, Petra Liskova, Alison Hardcastle, Stephen Tuft, Nikolas Pontikos

**Affiliations:** 1 UCL Institute of Ophthalmology University College London London United Kingdom; 2 Moorfields Eye Hospital London United Kingdom; 3 Centre for Optometry & Vision Science Biomedical Sciences Research Institute Ulster University Coleraine United Kingdom; 4 Laboratory of Visual Physiology, Division of Vision Research, National Institute of Sensory Organs National Hospital Organization Tokyo Medical Center Tokyo Japan; 5 Department of Ophthalmology Keio University School of Medicine Tokyo Japan; 6 Section of Ophthalmology School of Life Course Sciences King’s College London London United Kingdom; 7 Department of Twin Research and Genetic Epidemiology King’s College London London United Kingdom; 8 Department of Paediatrics and Inherited Metabolic Disorders, First Faculty of Medicine Charles University and General University Hospital Prague Czech Republic; 9 Department of Ophthalmology, First Faculty of Medicine Charles University and General University Hospital Prague Czech Republic

**Keywords:** artificial intelligence, machine learning, cornea, keratoconus, corneal tomography, subclinical, corneal imaging, decision support systems, corneal disease, keratometry

## Abstract

**Background:**

Keratoconus is a disorder characterized by progressive thinning and distortion of the cornea. If detected at an early stage, corneal collagen cross-linking can prevent disease progression and further visual loss. Although advanced forms are easily detected, reliable identification of subclinical disease can be problematic. Several different machine learning algorithms have been used to improve the detection of subclinical keratoconus based on the analysis of multiple types of clinical measures, such as corneal imaging, aberrometry, or biomechanical measurements.

**Objective:**

The aim of this study is to survey and critically evaluate the literature on the algorithmic detection of subclinical keratoconus and equivalent definitions.

**Methods:**

For this systematic review, we performed a structured search of the following databases: MEDLINE, Embase, and Web of Science and Cochrane Library from January 1, 2010, to October 31, 2020. We included all full-text studies that have used algorithms for the detection of subclinical keratoconus and excluded studies that did not perform validation. This systematic review followed the PRISMA (Preferred Reporting Items for Systematic Reviews and Meta-Analyses) recommendations.

**Results:**

We compared the measured parameters and the design of the machine learning algorithms reported in 26 papers that met the inclusion criteria. All salient information required for detailed comparison, including diagnostic criteria, demographic data, sample size, acquisition system, validation details, parameter inputs, machine learning algorithm, and key results are reported in this study.

**Conclusions:**

Machine learning has the potential to improve the detection of subclinical keratoconus or early keratoconus in routine ophthalmic practice. Currently, there is no consensus regarding the corneal parameters that should be included for assessment and the optimal design for the machine learning algorithm. We have identified avenues for further research to improve early detection and stratification of patients for early treatment to prevent disease progression.

## Introduction

### Background

Keratoconus is a bilateral ectatic disease of the cornea that can cause visual loss through corneal distortion and scarring [1,2]. The prevalence of keratoconus varies from 1 in 375 people in Northern Europe [3] to as high as 1 in 48 in some ethnic groups [4,5], with studies suggesting a higher incidence in Middle-Eastern, West Indian, and Asian populations with faster progression [6-8]. The onset of the disease typically occurs after puberty, with subsequent progression at a variable rate over 2 to 3 decades [6]. A recent meta-analysis found that patients <17 years are likely to progress more than 1.5 D in K_max_ over 12 months, and those with steeper K_max_ of more than 55 D are likely to have at least 1.5 D K_max_ progression [6].

As the disease advances, corneal distortion can reach a stage where spectacle-corrected vision is inadequate, and patients must rely on soft or rigid contact lenses to achieve good functional vision [9]. However, contact lenses are not always tolerated, and visual impairment can severely affect quality of life [10,11]. In the natural course of the disease, approximately 20% of the patients are offered a corneal transplant to improve their vision but at the risk of postoperative complications (eg, microbial keratitis and inflammation), potential allograft rejection, and transplant failure [7,12,13]. Most individuals with keratoconus are identified because of the symptoms of visual disturbance or an increase in astigmatism at refraction. Therefore, it is inevitable that most individuals with keratoconus are detected at a stage when visual deterioration has already occurred [14].

The detection of keratoconus at an earlier stage has become increasingly relevant since the introduction of corneal collagen cross-linking (CXL). This is a photochemical treatment of the cornea with UV-A light following the application of riboflavin (vitamin B2), which can arrest the progression of keratoconus in 98.3% of the eyes even in relatively advanced cases [15-20]. The benefit of early treatment to minimize visual loss is clear, and there is evidence that it is cost-effective [21-23], but the mechanism to improve early diagnosis by community-based optometrists is challenging because asymptomatic patients with subclinical disease are unlikely to seek review [14]. Improved detection will probably require improved access or efficient community screening with expensive imaging equipment [24].

Machine learning is a branch of artificial intelligence centered on writing a software capable of learning from data in an autonomous fashion by minimizing a loss function or maximizing the likelihood [25]. It can be broadly classified as either supervised or unsupervised learning [26]. In supervised learning, the algorithm is trained with input data labeled with a desired output so that it can predict an output from unlabeled input data [27]. In comparison, in unsupervised learning, the algorithm is not trained using labeled data. Instead, the algorithm is used to identify patterns or clusters in the data [28]. When applied to the field of keratoconus detection, machine learning may be used to analyze a large number of corneal parameters that can be derived from corneal imaging as well as other clinical and biometric measures such as visual acuity and refraction to predict the disease [29]. It can also be applied directly to imaging data to work at the pixel level [30]. Deep learning, a specific branch of machine learning, uses artificial neural networks (NNs) with multiple layers to process input data [31]. It is particularly well suited to the segmentation or classification of corneal images [32]. Both machine learning and deep learning may facilitate superior diagnostic ability that, when implemented as automated screening tools, could result in significant advances in case detection, mitigating both the cost of new imaging hardware and the burden on ophthalmic health care professionals [33]. In addition, through unsupervised learning, it may be possible to discover previously unknown disease subtypes or features [34,35].

Unlike diabetic retinopathy, which uses a widely adopted diagnostic grading system (Early Treatment Diabetic Retinopathy Study) [36] and in which the diagnosis of early disease is based on the presence of discrete entities on the retina (eg, microaneurysms), the diagnostic grading of subclinical keratoconus has not yet reached the same level of consensus [37]. Frequently used grading systems such as Amsler-Krumeich [38] and ABCD [39] do not specifically include a grade for subclinical keratoconus. More detailed information about keratoconus grading systems is available in Multimedia Appendix 1 [37-43].

### Case Definition for Keratoconus

Several terms describe the early stage of keratoconus before vision is affected, including forme fruste keratoconus (FFKC), keratoconus suspect, subclinical keratoconus, and preclinical keratoconus. The most commonly used terms are FFKC and subclinical keratoconus, but there is no consensus on their definition [44]**.**

We have included all papers that contain an identifiable subgroup of eyes with any of the aforementioned definitions because of the overlap in the nomenclature and lack of evidence as to which, if any, pose a particular risk for progression to clinical keratoconus. We excluded papers that only consider eyes with established keratoconus.

### Objectives

The aim of this study is to critically evaluate the literature on the algorithmic detection of subclinical keratoconus and its equivalent definitions. Advanced keratoconus is relatively easy to diagnose clinically, such that developing machine learning algorithms to identify advanced disease has limited utility. Therefore, we directed this review to publications that have included detection of subclinical keratoconus because identifying these individuals would allow for early treatment with CXL to reduce the likelihood of disease progression and visual loss. We have structured our review both around the different types of available input data (parameters, indices, and corneal imaging systems) and the machine learning algorithms for keratoconus detection. In addition, we investigated the validation methodology within each study and assessed the potential for bias.

### Research Questions

Our specific research questions are as follows:

1. Research question 1: What input data types have been used within subclinical keratoconus detection algorithms and how have they performed?
2. Research question 2: What machine learning algorithms have been used for subclinical keratoconus detection and how have they performed?
3. Research question 3: How was algorithm validation handled among the selected manuscripts?

## Methods

### Search Strategy

We conducted a literature review of the evidence for the utility of machine learning applied to the detection of keratoconus published between January 1, 2010, and October 31, 2020. The PRISMA (Preferred Reporting Items for Systematic Reviews and Meta-Analyses) Statement 2009 criteria [45] was followed to search 4 bibliographic databases: MEDLINE, Embase, Web of Science, and Cochrane Library using keyword search on their title, abstract, and keywords. The review was not registered, and no protocol was prepared.

We used the following keyword search for literature review in bibliographic databases: ((keratoconus) OR (cornea* protru*) OR (cornea* ectasia)) AND ((algorithm) OR (machine learn*) OR (deep learn*) OR (artificial intelligence) OR (detect*) OR (diagnos*) OR (screen*) OR (examin*) OR (analys*) OR (investigat*) OR (identif*) OR (discover*) OR (interpret*) OR (test*))

### Inclusion and Exclusion Criteria

We included studies that investigated the detection of early keratoconus or included a subgroup of patients with early disease, as defined by one of the following terms: subclinical keratoconus, FFKC, preclinical keratoconus, suspected keratoconus, unilateral keratoconus (normal fellow eye), and asymmetric ectasia (normal fellow eye) and any definition considered equivalent to the aforementioned terms. The studies should have reported the performance of their model on a data set that was separate from the training data set (often called a validation or a test set). This includes splitting of the data set into training and test sets (eg, 70% training and 30% testing), K-fold cross-validation (an extension of simple splitting, but the process is repeated K times, eg, when *K*=10, partition the data set into 90% for training the model and 10% for testing, and the process is repeated 10 times by choosing a different 10% partition each time for testing), or evidence of a validation study where the aim is to assess a previously derived model on a new data set (also known as an external validation). Finally, the full-text article should be available, and only papers published in English were considered.

We excluded papers based on the detection of early keratoconus defined as Amsler-Krumeich stages 1 or 2, as this represents established keratoconus with both clinical and topographical features [46].

### Data Synthesis

**Figure 1:**
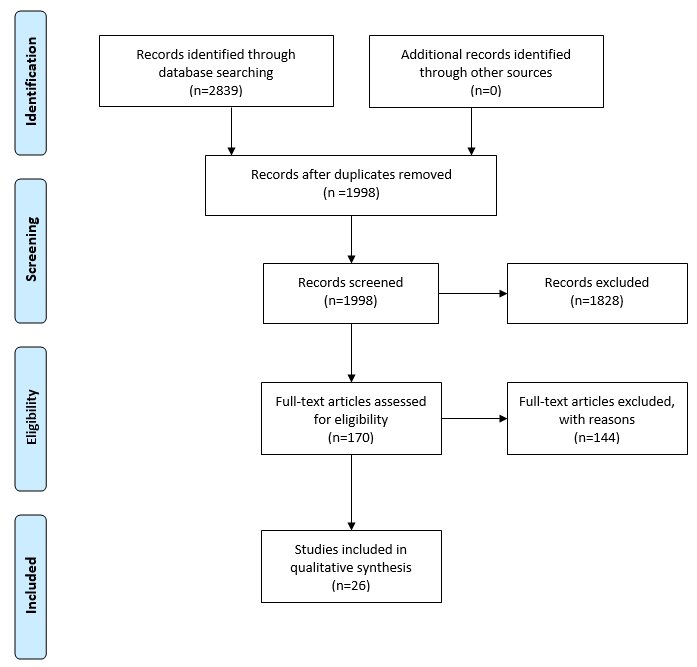
Filtering steps taken to accept or exclude studies in the systematic review.

On the basis of the inclusion criteria, 2 reviewers (HM and JPOL) screened the initial results. These results were then screened for the exclusion criteria by HM and NP. The PRISMA diagram is presented in Figure 1. Any disagreements in meeting the inclusion or exclusion criteria were resolved by discussion. Once the set of articles was finalized, 2 reviewers (HM and JPOL) analyzed each article and extracted the following information in a master table presented in Multimedia Appendix 2 [14,47-71]: author and year, title, system, sample source, country, age, gender, number of eyes for each group, diagnosis details, validation details, input details, input types, method, classification groups, sensitivity, specificity, accuracy, precision, area under the receiver operating characteristic curve (AUC), and source code availability. We summarized the most important information for all the results in Table 1. The main effect measures sought were sensitivity and specificity. If these statistics were not directly available from the article, they were calculated manually using their standard definitions [72]. To visually compare the results, we plotted the sensitivity and specificity across all studies for diagnostic criteria and detection systems in Multimedia Appendix 3.

**Table 1:** Summary of the 26 published studies that included the use of machine learning for the detection of subclinical keratoconus.

Study	System	Number of eyes		Fellow eye^a^	Input types	Method	Results (%)	
		Normal	Subclinical keratoconus				Sensitivity	Specificity
Arbelaez et al [47]	Sirius	1259	426	No	Elevation, keratometry, pachymetry, and aberrometry	SVM^b^	92	97.7
Saad et al [48]	Orbscan	69	34	No	Pachymetry, keratometry, elevation, and Displacement	DA^c^	92	96
Smadja et al [49]	GALILEI	177	47	Yes	Keratometry, pachymetry, elevation, aberrometry, demographic, and indices	DT^d^	93.6	97.2
Ramos-Lopez et al [50]	CSO topography system	50	24	No	Elevation and displacement	Linear regression	33	78
Cao et al [14]	Pentacam	39	49	No	Keratometry, pachymetry, and demographic	RF^e^, SVM, K-nearest neighbors, LoR^f^, DA, Lasso regression, DT, and NN^g^	94	90
Buhren et al [51]	Orbscan IIz	245	32	No	Keratometry, pachymetry, aberrometry, and elevation	DA	78.1	83.3
Chan et al [52]	Orbscan IIz	104	24	Yes	Pachymetry, keratometry, elevation, and displacement	DA	70.8	98.1
Kovacs et al [53]	Pentacam	60	15	Yes	Keratometry, pachymetry, elevation, indices, and displacement	NN	90	90
Saad et al [54]	OPD-scan	114	62	Yes	Keratometry, aberrometry, and indices	DA	63	82
Ruiz Hidalgo et al [55]	Pentacam HR	194	67	No	Keratometry, pachymetry, and aberrometry	SVM	79.1	97.9
Ruiz Hidalgo et al [56]	Pentacam HR	44	23	No	Keratometry, pachymetry, and indices	SVM	61	75
Xu et al [57]	Pentacam HR	147	77	Yes	Pachymetry, elevation, and keratometry	DA	83.7	84.5
Ambrosio et al [58]	Pentacam+Corvis ST	480	94	Yes	Pachymetry, elevation, keratometry, and Biomechanical	RF, SVM, and LoR	90.4	96
Sideroudi et al [59]	Pentacam	50	55	No	Keratometry	LoR	91.7	100
Francis et al [60]	Corvis ST	253	62	Yes	Biomechanical	LoR	90	91
Yousefi et al [61]	SS-1000 CASIA	1970	796	No	Elevation, pachymetry, and aberrometry	Unsupervised	88	14
Lopes et al [62]	Pentacam HR	2980	188	Yes	Pachymetry, elevation, indices, and displacement	DA, SVM, naive Bayes, NN, and RF	85.2	96.6
Steinberg et al [63]	Pentacam+Corvis ST	105	50	Yes	Pachymetry, elevation, keratometry, and biomechanical	RF	63	83
Issarti et al [64]	Pentacam	312	90	Yes	Elevation and pachymetry	NN	97.8	95.6
Chandapura et al [65]	RCTVue+Pentacam	221	72	Yes	Keratometry, elevation, pachymetry, aberrometry, and indices	RF	77.2	95.6
Xie at al [66]	Pentacam HR	1368	202	No	Heat maps	CNN^h^	76.5	98.2
Kuo et al [67]	TMS-4+Pentacam+Corvis ST	170	28	No	Heat maps	CNN	28.5	97.2
Shi et al [68]	Pentacam+ultrahigh resolution optical coherence tomography	55	33	Yes	Keratometry, elevation, pachymetry, indices, and demographic	NN	98.5	94.7
Toprak et al [69]	MS-39	66	50	Yes	Keratometry, pachymetry, and displacement	LoR	94	98.5
Issarti et al [70]	Pentacam HR	304	117	Yes	Elevation and Pachymetry	NN	85.2	70
Lavric et al [71]	SS-1000 CASIA	1970	791	No	Keratometry, pachymetry, and aberrometry	25 machine learning methods compares	89.5	96

^a^Fellow eye indicates whether the study defined subclinical keratoconus as the fellow eye of an individual with apparently unilateral keratoconus, with no clinical or topographical features of keratoconus.

^b^SVM: support vector machine.

^c^DA: discriminant analysis.

^d^DT: decision tree.

^e^RF: random forest.

^f^LoR: logistic regression.

^g^NN: neural network.

^h^CNN: convolutional neural network.

### Bias Assessment

When assessing bias within the included studies, we used a tailored version of the QUADAS (Quality Assessment of Diagnostic Accuracy Studies)-2 tool [73], which consists of 4 domains: patient selection, index test, reference standard, flow, and timing. The 26 studies were assessed by 3 reviewers (HM, JPOL, and NP) such that each study was assessed by at least 2 reviewers.

## Results

### Overview

**Figure 2:**
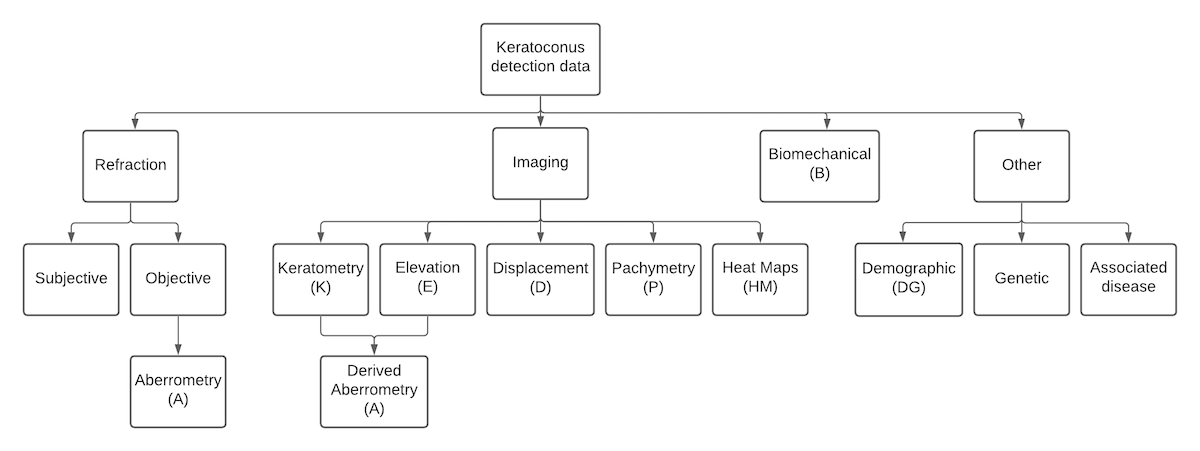
Organizational diagram of relevant data types reported to be used for the detection of subclinical keratoconus.

**Figure 3:**
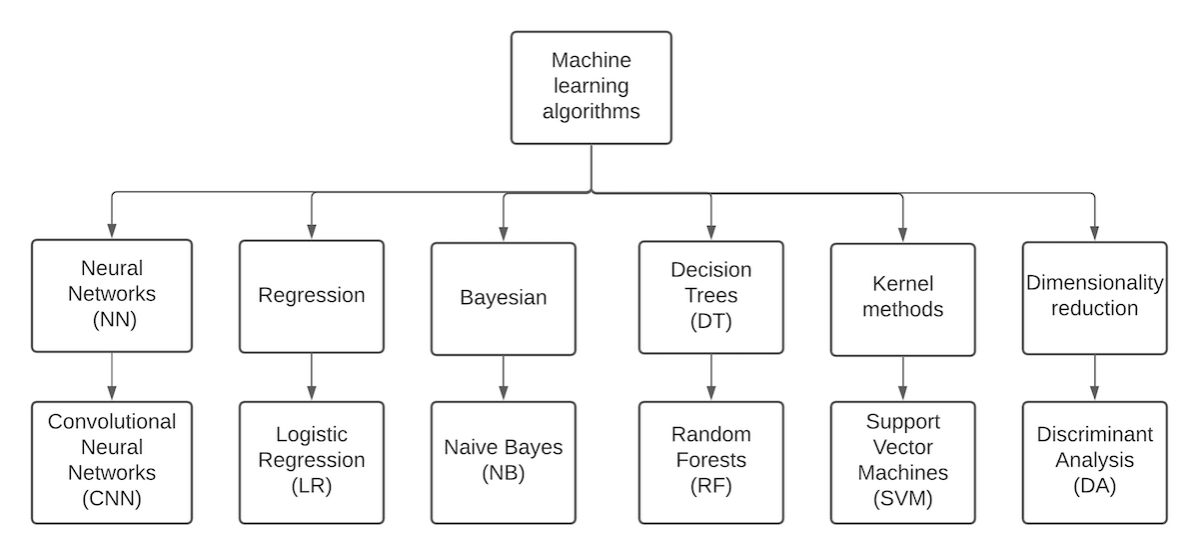
Organizational diagram of relevant machine learning algorithms used for the detection of subclinical keratoconus.

We identified 1998 potentially relevant papers published between 2010 and 2020. After filtering, we included 26 articles in our qualitative analysis. Table 1 summarizes these results, and a more extensive version can be found in Multimedia Appendix 2. To address research question 1, the results are discussed in terms of their input data. Charts displaying aggregate sensitivity and specificity can be found in Multimedia Appendix 3. To address research question 2, the results are considered in terms of the machine learning algorithms. Figures 2 and 3 present organizational diagrams of data categorization and machine learning algorithms, respectively. To maintain consistency, we opted to use the term *subclinical keratoconus* throughout regardless of the nomenclature used by the original authors. The original term is included in parenthesis, and details of the exact definition can be found in Multimedia Appendix 2.

### Research Question 1: What Input Data Types Have Been Used Within Subclinical Keratoconus Detection Algorithms and How Have They Performed?

This section is subdivided according to the input data types used for the detection of subclinical keratoconus, as presented in the organizational chart in Figure 2.

#### Aberrometry

Aberrometry was used to detect subclinical keratoconus in 31% (8/26) of the papers [47-49,51,55,61,65,71]. Aberrations are produced by imperfections in the optical quality of the refracting surface of the eye, including the cornea and the lens. Higher-order aberrations (HOAs) are measured from the distortion of a plane wavefront of light passing through the optics of the eye. However, HOAs can also be derived indirectly from the measurement of any distortion (eg, elevation) of the corneal surfaces. They can be described as a set of Zernike polynomials or with Fourier analysis. Using the Zernike method, aberrations can be subclassified as lower-order aberrations and HOAs. Lower-order aberrations include simple defocus (myopia or hyperopia) and regular astigmatism, which account for approximately 90% of the refractive error of the normal eye [74]. The most clinically relevant HOAs are spherical aberration, coma, and trefoil that cannot be corrected by glasses or a soft contact lens. In keratoconus, the irregular distortion of the front and back surfaces of the cornea causes visually significant HOAs. Arbelaez et al [47] analyzed these parameters in their subclinical keratoconus detection model and included a weighted sum of HOAs (known as the Baiocchi-Calossi-Versaci index) and the root mean square of HOAs. Moreover, 5 other studies also used derived Zernike aberrometry data [48,49,51,65,71]**.**

#### Corneal Imaging Data and Derived Parameters

##### Overview

**Figure 4:**
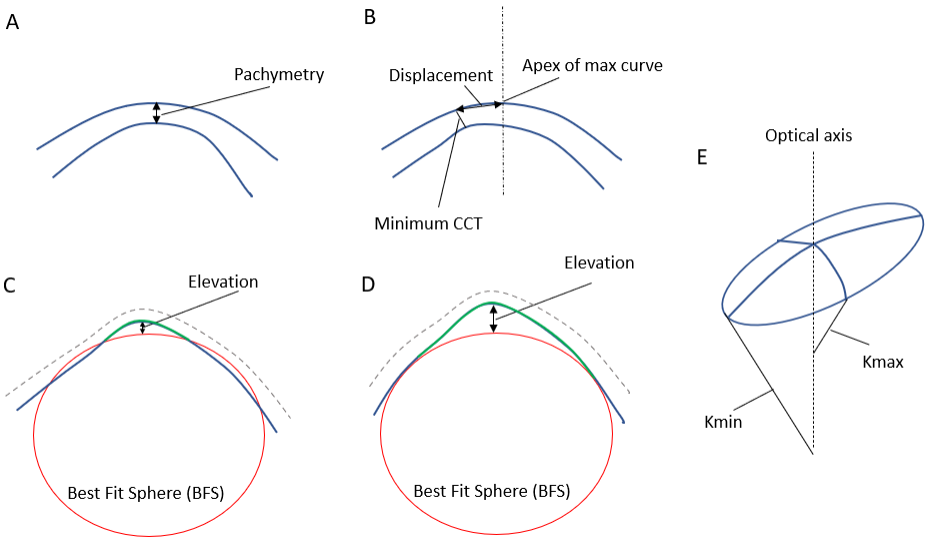
Schematic diagram illustrating the 4 basic corneal parameters that can be measured using corneal imaging. (A) pachymetry. (B) displacement: distance between the apex of the cornea and the point of minimum thickness. (C) and (D) represent 2 methods of calculating the best-fit sphere (BFS). In (C) the BFS is fitted to both the normal peripheral posterior surface (blue) and the abnormal anterior protrusion of the central posterior surface (green). In (D) the BFS is fitted to only the normal peripheral posterior surface (blue) excluding the abnormal central posterior surface (green), leading to a larger relative elevation than in (C). (E) the smallest radius of curve of the astigmatic corneal surface corresponds to the largest refractive power (Kmax) and the largest radius of curve corresponds to the smallest refractive power (Kmin). CCT: central corneal thickness.

**Figure 5:**
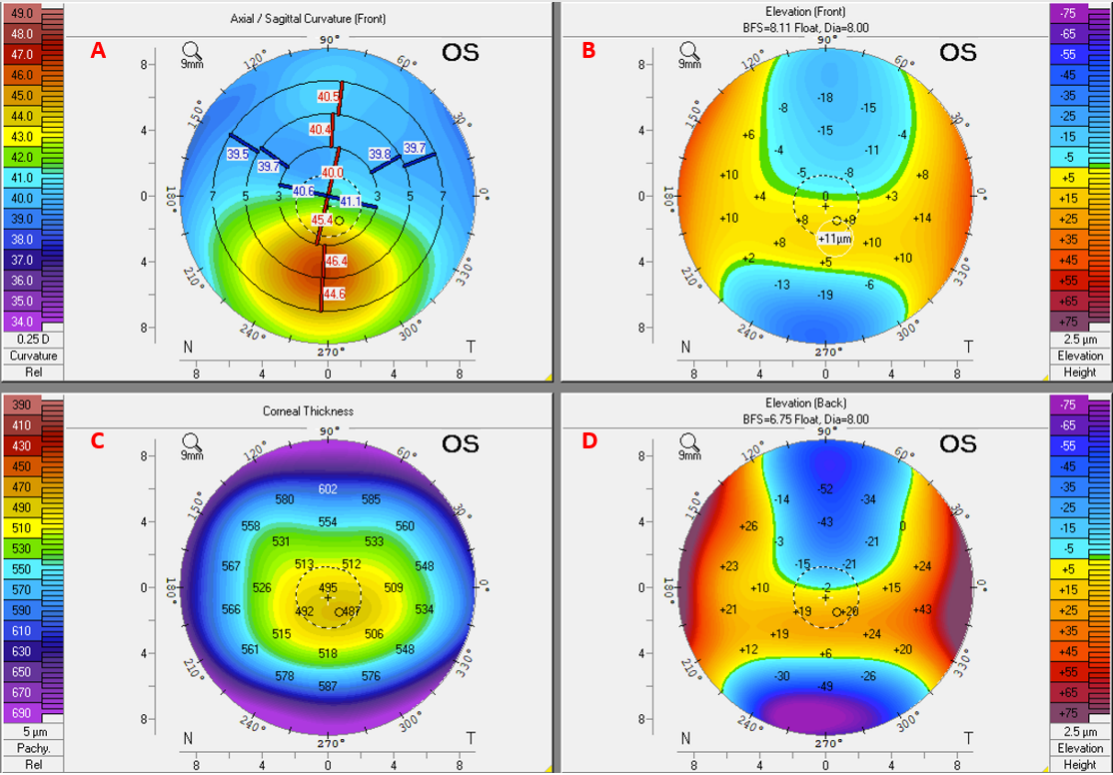
Heat maps of a subclinical keratoconus eye derived from Scheimpflug corneal imaging using the Pentacam HR device. The axial/sagittal map (A) depicts the curvature of the anterior corneal surface in dioptres and shows mild inferior steepening, while the pachymetry map (C) shows thinning in the same region. The front and back elevation maps (B and D, respectively) show a moderate increase in inferior elevation. BFS: best-fit sphere; OS: left eye.

Corneal images were used to detect subclinical keratoconus in 96% (25/26) of the papers. There are various acquisition techniques, including Scheimpflug optics (Pentacam [Oculus GmbH] or Sirius [CSO]), anterior segment optical coherence tomography (AS-OCT; MS-39 [CSO], or CASIA [Tomey]), and horizontal slit-scanning systems such as Orbscan II (Bausch & Lomb). These systems incorporate a software that processes the images to derive numerical indices or secondary images, such as heat maps, to visualize various aspects of corneal shape. These parameters can be classified as measurements of the corneal surface radius of curvature (keratometry), elevation or depression of a point on the corneal surface from the mean (elevation map), corneal thickness (pachymetry), or displacement from the apex of the cornea. Figure 4 illustrates the main parameter types in a schematic diagram. Figure 5 shows an example of the Pentacam heat map for an eye with subclinical keratoconus. See Multimedia Appendix 4 for an example of advanced keratoconus (fellow eye for the same patient).

In the following subsections, we briefly discuss the use of quantitative measures derived from corneal imaging when used in isolation or in combination with machine learning models.

##### Keratometry Parameters

Keratometric data are one of the most commonly used parameters in the literature, with 69% (18/26) of the papers incorporating keratometry as one of the parameters in their model [14,47-49,51-54,56,57,59,61,65,68,71,75]. Keratometric parameters measure the radius of curvature of the anterior or posterior corneal surfaces. Examples include the meridian with the minimum corneal radius of curvature (corresponding to K_max_) and maximum curvature (corresponding to K_min_). When looking at individual keratometric parameters derived using Fourier analysis for subclinical keratoconus detection, Sideroudi et al [59] achieved a predictive accuracy of over 90% using higher-order irregularities, asymmetry, and regular astigmatism, primarily in the corneal periphery.

##### Elevation Parameters

Overall, 62% (16/26) of the papers incorporated elevation parameters in their analysis [47-53,57,58,61-65,68,70]. Elevation represents points above or below the BFS of the corneal surface measured in microns (Figure 4). For the posterior cornea, this is measured either as the divergence from the best fit of the whole posterior corneal diameter or as the divergence from the best fit of the annulus of the peripheral posterior corneal surface outside the central 4 mm [76]. The latter method, the Belin-Ambrosio map, better describes the central corneal elevation, which is a feature of keratoconus. Values can be presented as either color-coded maps or individual parameters such as maximum anterior elevation, maximum posterior elevation, or derived data such as aberrometry.

Posterior corneal curvature consistently outperforms other parameters in the discrimination of subclinical keratoconus [47,49,56,57]. Its inclusion increases the sensitivity of a support vector machine (SVM) from 75.2% to 92% and precision from 57.4% to 78.8% but has a limited impact on specificity [47]. Posterior corneal curvature, measured using a Pentacam (Scheimpflug) device and analyzed using an SVM, was also found to be an important parameter for sensitivity and much less so for specificity and AUC [14]. Similarly, using the Galilei (Scheimpflug) device, the posterior asphericity asymmetry index was found to be the variable with the most discriminatory power when differentiating normal from subclinical keratoconus, followed by corneal volume [49]. Conversely, analysis of anterior surface topographical parameters and aberrometry using the random forest algorithm did not discriminate subclinical keratoconus (very asymmetric ectasia-normal topography) from normal eyes [65].

Saad et al [54] showed that combining parameters obtained from the anterior corneal curvature corneal wavefront and Placido-derived indices lead to a better discriminative ability between normal and subclinical keratoconus eyes (FFKC) over a Placido-only–based algorithm.

##### Displacement Parameters

A total of 23% (6/26) of the papers used displacement parameters in their analysis [48,50,52,53,62,75]. These represent measures such as the displacement of the point of minimum corneal thickness from the corneal apex. Of these, 3 papers used the displacement of the thinnest point from the geometric center of the cornea in their model [48,52,77]. Kovacs et al [53] used the vertical and horizontal decentration of the thinnest point and found them to be the best parameters to discriminate normal fellow eyes of keratoconus from control eyes using an NN.

##### Pachymetry Parameters

Overall, 77% (20/26) of the papers used pachymetry data in their model, making it one of the most commonly used parameters in the literature [14,47-49,51-53,55-58,61-65,69-71]. Pachymetry is the thickness of the cornea, measured using either ultrasound or imaging techniques. Simple examples include central corneal thickness and the thinnest point of the cornea. A reduction in the thickness of the central cornea is a fundamental biomarker of keratoconus [2].

##### Summary Indices

In total, 23% (6/26) of the papers used summary indices in their model [14,49,53,65,68,78]. In addition to single-parameter measurements (eg, central corneal thickness), tomographic systems such as the Pentacam can combine measurements to compute derived indices that estimate the regularity of corneal shape. Basic indices such as the index of surface variance, index of vertical asymmetry, or index of height asymmetry are formed from multiple data points. Composite indices are formed from other indices and data points. Examples include the Keratoconus index, keratoconus percentage index, and Belin/Ambrosio enhanced ectasia display (BAD-D). In a recent study, Shi et al [68] used 6 indices from the Pentacam along with keratometric, elevation, and pachymetric parameters derived from the Pentacam and ultrahigh resolution optical coherence tomography to create an NN classifier to discriminate between normal and subclinical keratoconus eyes. Using 50 normal eyes, 38 eyes with keratoconus, and 33 eyes with subclinical keratoconus, they achieved 98.5% sensitivity and 94.7% specificity. However, the results require further validation because of the small number of eyes in this group. Furthermore, the authors did not include a comparison between existing detection metrics, such as BAD-D.

##### Heat Maps

A total of 8% (2/26) of the papers used heat maps in their detection model [66,67]. Modalities such as Scheimpflug and AS-OCT capture images at various corneal meridians and subsequently use these data to derive the heat maps that facilitate visual interpretation of the data, although there is extrapolation of the data in areas between the imaged meridians. For example, the Pentacam can translate the raw images into several types of color heat maps (eg, axial curvature, posterior or anterior elevation, and regional pachymetry) based on the same original tomography data set. Prediction models applied to images often use convolutional NNs (CNNs), and studies applying these methods are discussed in detail in the next section addressing research question 2. To the best of our knowledge, no system has used raw pixel values from Scheimpflug or AS-OCT images directly when detecting subclinical keratoconus.

#### Biomechanical Data

Overall, 12% (3/26) of the papers incorporated biomechanical data in their analysis [58,60,63]. Corneal biomechanics refers to the distortion response of the cornea to an applied force. The Ocular Response Analyzer (Reichert Ophthalmic Instruments) uses a puff of air directed to the cornea, and the deformation response is measured. Two common indices have been reported: corneal hysteresis and corneal resistance factor. However, there is disagreement regarding their utility in the diagnosis of keratoconus [79,80]. Another device using the same principle is the Corvis ST (Oculus Optikgeräte GmbH), which uses a high-speed Scheimpflug camera to measure distortion in cross-sectional images. Numerous studies have described the application of machine learning to analyze biomechanical data, but very few validated their results; therefore, they have been excluded from this review. Ambrosio et al [58] combined Pentacam and Corvis ST data to create the Tomographic and Biomechanical Index, and this was followed up with a validation study [63]. Francis et al [60] used biomechanical data from the Corvis ST device when diagnosing keratoconus and achieved very high sensitivity (99.5%) and specificity (100%). However, when validating their model, they have only discriminated between 2 groups—a group combining subclinical keratoconus and keratoconus eyes, and a group of normal eyes. This represents an easier problem than including a distinction between normal and subclinical keratoconus eyes.

#### Demographic Risk Factors

A total of 15% (4/26) of the papers chose to include demographic data, such as age or sex, in their model [14,49,62,68]. Cao et al [14] demonstrated that sex was an important parameter in a minimum set that achieved the highest AUC using the random forest method, although their data set was small (49 subclinical keratoconus and 39 control eyes). Ethnicity, a major association with disease prevalence, risk of progression, disease severity, and acute corneal hydrops in Asian and Black populations [81], was not included in any model, although some studies have examined single ethnicities [66]. Ethnicity as a parameter should be considered by future investigators. No studies included other risk factors such as atopy and eye rubbing as model parameters, and these should be considered in future studies.

### Research Question 2: What Machine Learning Algorithms Have Been Used for Subclinical Keratoconus Detection and How Have They Performed?

In most cases, researchers have used combinations of parameters and indices within machine learning algorithms to diagnose subclinical keratoconus. This section is subdivided according to the machine learning techniques that were applied. Figure 3 presents an organizational diagram of the relevant machine learning algorithms. There are several other algorithms, but discussion of these is beyond the scope of the review, and we have chosen to include only the methods found in our *Results* section.

#### Neural Networks

##### Overview

NNs consist of a series of interconnected layers of neurons and are thus loosely modeled on the structure within the human brain. Each neuron computes a nonlinear function of its inputs, and the network is trained until the output aligns optimally with the ground truth labels. Kovacs et al [53] used a combination of 15 keratometric, pachymetric, and elevation parameters in an NN classifier to discriminate healthy corneas from fellow eyes of patients with unilateral keratoconus. The patient data included 60 normal eyes from 30 patients, 60 bilateral keratoconus eyes from 30 patients, and 15 normal eyes from patients presenting with unilateral keratoconus. When classifying the normal eyes of the patients with unilateral keratoconus with clinical grading as a reference, they achieved 90% sensitivity and 90% specificity. They took a novel approach of training on both the eyes of the patients, which allowed them to incorporate the effect of any intereye asymmetry when detecting unilateral keratoconus. Shi et al [68] combined keratometric, elevation, and pachymetric parameters derived from Pentacam images and ultrahigh resolution optical coherence tomography to create an NN classifier for discriminating normal from subclinical keratoconus eyes. Using Pentacam elevation and pachymetry maps within a hybrid NN model, Issarti et al [64] demonstrated superiority over other common diagnostic indices such as BAD-D and topographical keratoconus classification.

##### Convolutional Neural Networks

When images are used for analysis, NNs with a large number of processing layers such as CNNs are often employed because of their ability to make inferences from 2D or 3D data structures through deep learning [82]. For example, Xie et al [66] used data from 1368 normal eyes, 202 eyes with early keratoconus, 389 eyes with more advanced keratoconus, and 369 eyes with subclinical (suspected) keratoconus to develop an automatic classifier. They achieved 76.5% sensitivity and 98.2% specificity when classifying subclinical keratoconus. However, the heat maps used were produced by Pentacam; therefore, it should be noted that the technique may not be transferable to other systems or even future Pentacam software iterations. Kuo et al [67] included 150 normal, 170 keratoconus, and 28 subclinical eyes in their study and used the Tomey TMS-4 topography system to produce corneal heat maps and trained 3 different CNN architectures (VGG16, InceptionV3, and ResNet152). When attempting to identify the 28 subclinical keratoconus eyes, they applied the VGG16 model and achieved *barely satisfactory* results with an accuracy of 28.5% when a threshold of 50% was applied. These results suggest that subclinical keratoconus cannot yet be detected with high sensitivity using CNNs on heat map images.

#### Decision Trees

The classification of data in a decision tree uses a binary decision at each node in the tree to determine the branch to take next. Starting from the root, the classification is determined by following each branch to its terminal node. Smadja et al [49] used a decision tree to classify normal, keratoconus, and subclinical (FFKC) keratoconus eyes. They enrolled 177 normal eyes, 148 keratoconus eyes, and 47 subclinical eyes. They used 55 parameters (including curvature, elevation, corneal wavefront, corneal power, pachymetry, and age) collected from the Galilei dual Scheimpflug camera, achieving 93.6% sensitivity and 97.2% specificity when classifying subclinical from normal. Cao et al [14] also evaluated a decision tree algorithm for classifying subclinical keratoconus but achieved lower sensitivity (82%) and specificity (78%). They attributed the comparatively inferior performance to the fact that Smadja et al [49] used additional machine-specific indices that they did not have access to.

##### Random Forests

Random forests combine a large number of decision trees into a single model [83]. Lopes et al [62] compared this method with other methods (naive Bayes, NNs, SVMs, and discriminant analysis) by training models on 71 post–laser-assisted in situ keratomileusis (LASIK) ectasia eyes, 298 post-LASIK eyes without ectasia, and 183 eyes with keratoconus. They included keratometry, pachymetry, elevation, and various Pentacam indices. The models were validated on an external data set containing 298 normal eyes (stable LASIK), 188 keratoconus eyes (very asymmetric ectasia-ectatic), and 188 subclinical eyes (very asymmetrical ectasia-normal topography). The latter 2 groups were collected from the same set of patients. They found that the random forest model performed best when detecting subclinical eyes with an 85.2% sensitivity. This accuracy is lower than that of other comparable studies, which is probably caused by their inclusion of external validation rather than an inferior model. The authors also note that their model classifies among 3 groups, whereas other related studies (such as that by Arbelaez et al [49]) only classify between 2 groups (eg, subclinical vs normal). This important distinction is expanded upon in the *Discussion* section.

#### Discriminant Analysis

Discriminant analysis uses a linear combination of variables that optimally separate 2 or more classes of data. Xu et al [57] used this method to classify eyes as either normal, subclinical keratoconus, or keratoconus. In total, 147 normal eyes, 139 eyes with keratoconus, and 77 eyes with subclinical keratoconus were included in the training set and verified on a separate set of 97 normal and 49 subclinical keratoconus eyes. They applied the Zernike fitting method to corneal pachymetry and elevation data derived from the Pentacam and achieved an AUC of 92.8% when discriminating subclinical keratoconus. Saad et al [54] also used discriminant analysis to classify eyes as either subclinical (FFKC) keratoconus or normal. They used a combination of wavefront aberrometry and Placido disc indices in their model with a total of 8 parameters using the OPD-Scan (Nidek Co Ltd). The model was trained on 114 normal and 62 subclinical eyes and validated on 93 normal and 82 subclinical eyes. Using training data only, the model achieved 89% sensitivity and 92% specificity, but when applied to the validation set, the accuracy dropped significantly to 63% sensitivity and 82% specificity. This highlights the need for external validation when reporting the performance of the detection algorithms.

#### Support Vector Machines

SVMs translate data into a higher-dimensional space where a dividing line (known as a hyperplane) separates the data such that the distance between the hyperplane and any given data point is maximized [26]. When 8 different machine learning algorithms were compared for classifying subclinical keratoconus on the same data set, SVMs achieved the highest sensitivity (94%) [14]. Arbelaez et al [47] achieved even higher sensitivities using SVMs with a large data set of 1259 normal eyes and 426 with subclinical keratoconus. They used 200 eyes from each group for training and the remainder for testing, achieving 92% sensitivity and 97.7% specificity. Ruiz Hidalgo et al [56] used 25 topographic or tomographic Pentacam-derived parameters to verify their SVM model. They included 131 patients in their study and provided results for 2 classifications from separate hospitals: Antwerp University Hospital and Rothschild Foundation, Paris. When classifying the 4 groups (keratoconus, subclinical, normal, and postrefractive surgery), the sensitivity for subclinical keratoconus detection was 61% compared with that of the Antwerp University Hospital classification and 100% compared with that of the Rothschild classification. This was a comprehensive validation study that compared multiple methods with 2 subjective reference standards. Only a small number of subclinical keratoconus cases (approximately 20) were included in this study, and a larger study is required to verify these results.

#### Logistic Regression

Logistic regression is commonly used to perform classification from a set of independent variables [26]. It transforms its output using the sigmoid function to return a probability that can then be thresholded for classification. When classifying subclinical keratoconus, 3 studies used this technique exclusively [59,60,75]. Sideroudi et al [59] used logistic regression to explore the diagnostic capacity of Fourier-derived posterior keratometry parameters (spherical component, regular astigmatism, asymmetry, and irregular astigmatism) extracted from Pentacam Scheimpflug images. They included 50 normal eyes, 80 eyes with keratoconus, and 55 with subclinical keratoconus (defined as a clinically normal eye with abnormal topography, where the fellow eye has advanced keratoconus) and validated their model on 30% of the data set. Their model attained 91.7% sensitivity and 100% specificity when classifying between subclinical keratoconus and normal eyes. Although these results are among the best reported, the study has yet to be validated using an external data set. Other studies implemented logistic regression as part of a wider comparison of machine learning algorithms [14,58].

#### Comparative Studies

Few studies have applied multiple machine learning algorithms to the same data set. Cao et al [14] tested 8 machine learning algorithms on the same data set of 39 normal control eyes and 49 eyes with subclinical keratoconus. Age, sex, and 9 corneal parameters from the Pentacam tomography were used, and the authors found that random forest, SVM, and K-nearest neighbors had the best performance. Random forests had the highest AUC of 0.97, SVM had the highest sensitivity (94%), and K-nearest neighbors had the best specificity (90%). Although they verified their results with 10-fold cross-validation, it would be instructive to repeat the analysis on a larger external data set. Ambrosio et al [58] also performed an analysis across algorithms including logistic regression, SVMs, and random forests to classify between 4 groups: normal, keratoconus, very asymmetrical ectasia-ectatic, and subclinical keratoconus (very asymmetric ectasia-normal). They used both Scheimpflug tomography and biomechanical data and included 480 normal eyes, 204 eyes with keratoconus, 72 eyes classified as very asymmetrical ectasia-ectatic, and 94 subclinical keratoconus eyes. When considering subclinical keratoconus, the random forest model performed the best, with 90.4% sensitivity and 96% specificity. The final model was named the Tomography and Biomechanical Index and was validated by leave-one-out cross-validation, resulting in as many models as there were subjects (N=850). Lopes et al [62] also performed a comparative analysis and found that random forests performed best when trying to classify 3 groups of eyes (including subclinical eyes). Lavric et al [71] provided the largest comparative study for detecting subclinical keratoconus. The authors included 1970 normal eyes, 390 eyes with keratoconus, and 791 subclinical (FFKC) keratoconus eyes in their study and used keratometric, pachymetric, and aberrometric data from the CASIA AS-OCT system in their analysis across 25 different machine learning algorithms. When they classified the 3 groups simultaneously, they found that the most accurate method was SVM, which attained 89.5% sensitivity for the detection of subclinical keratoconus, and the results were validated using 10-fold cross-validation. The limitations of this study include the use of the CASIA ectasia screening index (ESI) for the classification of the severity of keratoconus, which may not agree with clinical diagnosis, and that the analyzed parameters are closely tied to the CASIA device, which limits generalizability to other systems.

#### Unsupervised Learning

Unsupervised learning represents a distinct approach to the detection of subclinical keratoconus by attempting to identify groups of similar eyes without prelabeled data. Yousefi et al [61] used a 2-step approach that combined dimensionality reduction and density-based clustering to cluster a cohort of 3156 eyes categorized according to the ESI index as either normal, keratoconus, and subclinical (FFKC) keratoconus. They included 420 topography, elevation, and pachymetry parameters, and the algorithm produced 4 clusters of eyes with similar characteristics. When comparing their results with a reference standard (ESI), the model did not create a distinct grouping that separated the subclinical eyes from other eyes (sensitivity 88% and specificity 14%), suggesting poor correlation when compared with ESI alone. Furthermore, they did not compare their results with clinically labeled data.

### Research Question 3: How Was Algorithm Validation Handled Among the Selected Manuscripts?

Although most studies performed internal validation by splitting the original data set into training and test sets, we identified 5 replication papers that validated a published model on a new data set [48,51,52,56,63]. Ruiz Hidalgo et al [56] verified their SVM technique presented in 2016 [55]. The authors found that when using the Antwerp University Hospital classification, there was approximately 18% decrease in sensitivity, whereas when using the Rothschild classification, there was approximately 21% increase in sensitivity. These discrepancies highlight the problems associated with subjective classification and the absence of ground truth. Furthermore, when multiple groups were included in the analysis; that is, normal, keratoconus, subclinical keratoconus, and postrefractive surgery eyes, it was noted that the accuracy decreased from 93.1% in discriminating normal from FFKC to 88.8%. However, this paper presented the most comprehensive methodology because the authors not only verified their results on a new sample population with multiple target classes but also compared their results with other methods and included 2 subjective reference standards.

Buhren et al [51] validated their model defined in 2010 [84]. When comparing their discriminant function derived from anterior and posterior corneal surface wavefront data, they reported approximately 22% decrease in sensitivity and approximately 9% decrease in specificity. This decrease was likely caused by overfitting in the original study. Saad et al [48] and Chan et al [52] validated the same discriminant analysis model presented by Saad et al [77]. Saad et al [48] reported sensitivity (92%) and specificity (96%), roughly in line with their previous study, which indicates that their method is reliable and does not suffer from overfitting. Chan et al [52] validated the original model in patients from different ethnic backgrounds (Asian). They reported approximately 21% decrease in sensitivity, which they attributed to overfitting in the original study; however, their specificity was almost equivalent. Steinberg et al [63] validated the work presented by Ambrosio et al [58]. They reported approximately 27% decrease in sensitivity and approximately 13% decrease in specificity when applying the same thresholds.

### Bias Assessment

In general, patient selection was found to have a high risk of bias (19/26, 73% of studies) because most studies were case-control (thus susceptible to selection bias) and did not use consecutive or random samples. Multimedia Appendix 5 [14,47-71] contains the results of applying the QUADAS-2 tool when considering the risk of bias. The index test was also generally found to have a high risk of bias (21/26, 81% of studies) because of the lack of external validation. As there is no gold standard for subclinical keratoconus diagnosis, we could not assess the bias for the reference standard; therefore, all papers were marked as unclear. Finally, patient flow was found to have a low risk of bias (21/26, 81% of studies) because although chronological information was sparse, the same analysis was usually applied to all patients.

## Discussion

### Research Question 1: What Input Data Types Have Been Used Within Subclinical Keratoconus Detection Algorithms and How Have They Performed?

The data most commonly used for building subclinical keratoconus detection algorithms are numeric keratometry or pachymetry parameters; hence, according to our review, algorithms based on these tend to have the highest performance. These parameters are derived from a variety of imaging systems and devices and are then incorporated into different combinations to build a classification system or an index. Inevitably, individual systems produce parameters that may not be comparable across devices, and for proprietary reasons, the raw data are generally not available to derive these parameters. Therefore, comparison or replication across systems is difficult. Heat maps provide a visual representation of either corneal elevation, pachymetry, or curvature, which are helpful for the visual interpretation of results. However, heat maps require interpolation or extrapolation of data, which may introduce inaccuracies when included in the model. To the best of our knowledge, there are no studies that have analyzed actual pixel-level corneal imaging data (Scheimpflug or AS-OCT), probably because access to these data is restricted to commercial machines such as the Pentacam, which impedes bulk export to train machine learning algorithms.

We also noted that many studies do not incorporate details of patient demographics and associated diseases, such as age, sex, ethnicity, and atopy, which can influence the risk of developing keratoconus. Incorporating these data into these models may help define the population to which an algorithm applies, particularly as there are phenotypic indices that an algorithm can identify from images that humans cannot identify by manual inspection [85].

### Research Question 2: What Machine Learning Algorithms Have Been Used for Subclinical Keratoconus Detection and How Have They Performed?

Subclinical keratoconus studies typically involve univariate or multivariate analyses. For univariate studies, receiver operating characteristic analysis is performed, as each parameter is included to quantify their diagnostic ability. However, because none of the univariate studies we identified performed an out-of-sample validation, they were all excluded. For multivariate studies, machine learning is used to create a detection model using multiple parameters. These algorithms have already demonstrated comparable performance to experienced ophthalmologists in the identification of retinopathy of prematurity [86] and retinal disease progression [87]. Machine learning–based research into the detection of subclinical keratoconus has largely focused on supervised learning techniques, such as decision trees, SVM, logistic regression, discriminant analysis, NNs, and CNNs. Logistic regression may be superior to NNs when parameters from a single imaging modality are considered [14,68], with a potentially greater role for NNs when a large number of potentially interacting parameters are combined, such as for multiple imaging modalities [68]. Unsupervised learning has also been evaluated for the detection of subclinical keratoconus, although it relies on identifying patterns in large amounts of data; hence, it may not translate to a different data set of a different size and with different properties. In addition, with the exception of the study by Yousefi [61], none of the papers provided access to the source code for their algorithms or a description of the hyperparameters, which makes it difficult to reproduce and validate the results with external data sets.

### Research Question 3: How Was Algorithm Validation Handled Among the Selected?

We excluded papers that did not include a validation arm for the study, and the vast majority of initially identified studies did not appropriately validate their results. For any type of automatic classifier, validating the results on a data set distinct from the trained set is critical in determining the generalizability of the model to other data sets. With the exception of the studies by Saad et al [48] and Hidalgo et al [56], it is clear that studies attempting to validate a prior method reported significant decreases in sensitivity and specificity in comparison with their original results. This shows that even when techniques such as cross-validation are performed, the best method for validation is an independent out-of-sample data set, and its absence may introduce bias. Ideally, this external data set would be larger and more representative of the general population.

### Strengths and Limitations

The primary strength of this study is that we present a comprehensive review of all studies published in English between January 1, 2010, and October 31, 2020, on the use of machine learning for the detection of subclinical keratoconus. Our focus on the detection of subclinical keratoconus addresses an important unmet clinical need for an effective machine-based technique to identify keratoconus at its earliest stage. This would move us closer to potential screening without significant demands on clinicians and clinical services. Subclinical disease diagnosis is more challenging than the detection of advanced disease, where the opportunity to prevent progression has already been lost. In this respect, our review builds on recent clinical trials of CXL to prevent keratoconus progression in children and young adults [15,88,89]. To present a balanced and comprehensive overview, we have combined the expertise of computer scientists (HM and NP) familiar with the development of machine learning for clinical medicine with the input from clinicians (JPOL, DG, and ST) who are experienced in keratoconus management. We have considered and compared the literature in terms of both clinical input data and machine learning methodology, which allows the reader to gain a wider perspective of the problem.

However, there are limitations to our search methods and inclusion criteria. As with any systematic review, articles that did not include the relevant key terms or were not appropriately indexed by the literature databases may have been missed. When considering our inclusion criteria based on subclinical disease, some studies may have been missed because of a lack of consensus on definition. In addition, where there was no form of validation, we excluded the study; thus, our results represent only the articles that have some degree of generalizability.

A further limitation is the difficulty in comparing the performance of the approaches described in the manuscripts; direct comparisons were not possible because of the variation of multiple study design factors such as subclinical disease definition, parameter choice, data set source, and machine learning algorithm. Finally, a limitation regarding case definition that applies to all studies is the uncertainty in the relationship between subclinical keratoconus and other nonprogressive abnormalities of corneal shape.

### Challenges and Future Directions

Our systematic review identified several challenges from the literature and avenues for future research.

#### Case Definition, Gold Standard, and Ground Truth

Precise comparisons between the results of publications are problematic because of the ambiguous definition of early keratoconus and the absence of a gold standard examination technique. The most common definition of subclinical keratoconus is an eye with topographic findings that is at least suspicious of keratoconus and with confirmed keratoconus in the fellow eye. FFKC is usually defined as an eye that has both normal topography and slit-lamp examination but with keratoconus in the fellow eye [44]. With this differentiation, subclinical keratoconus will be easier to detect than FFKC, and studies using the former definition are likely to produce more accurate results because the problem becomes easier to solve. The problems of making statistical comparisons in the absence of a gold standard have been discussed extensively by Umemneku et al [90]. The authors suggest that latent class analysis, composite reference standards, or expert panel analysis may be appropriate in these circumstances.

Even if a precise definition of early subclinical keratoconus was established, the absence of ground truth data is relevant when evaluating the precision of data acquisition. For example, measurements of keratoconus taken by different operators or repeated on different days may lead to variations in the results. Flynn et al [91] found that keratometric measurements from Scheimpflug images (Pentacam) were more reproducible in early keratoconus (mean central K ≤53 D) compared with those in more advanced keratoconus (mean central K>53 D), although a cohort with subclinical keratoconus was not included. In contrast, Yang et al [92] found that biomechanical parameters (Corvis ST) had acceptable repeatability in both normal and keratoconus eyes.

Another issue we identified when comparing studies was the variation in the number of groups that were classified. The studies often started with multiple groups (usually 3, eg, FFKC, keratoconus, and normal); however, 21 papers chose to report their accuracy results from a model trained to classify between just 2 groups (eg, FFKC and normal), whereas 5 papers reported results for classifying between all groups. Classifying all groups is a more realistic clinical scenario, but it presents a more challenging problem because the features of the different groups can overlap. Complete details of the number of groups associated with the accuracy results are presented in Multimedia Appendix 2.

#### Study Size and Statistical Power

The size of the study is critical when developing a reliable detection system. In particular, the accuracy of machine learning models is directly related to the amount of training data. When considering eyes with subclinical keratoconus, only 2 studies included more than 500 eyes [61,71]. None of the papers included a priori power calculations to estimate the size of the cohort to be studied.

#### Study Design

None of the reported studies evaluated the performance of their method against masked observers; thus, they may introduce detection bias. The initial classification is often made by considering the fellow eye with keratoconus as a factor in the decision-making process, whereas the algorithm does not have this information. Hence, it would be interesting to design a study where, having already decided on the ground truth diagnosis, a new clinician is asked to evaluate the eye using the same information as the algorithm (ie, only the images or parameters). This situation is closest to real-life screening where a prospective patient (without a history of keratoconus in either eye) is examined for risk of keratoconus.

Subclinical keratoconus is, by definition, the least affected eye of highly asymmetrical keratoconus. An assumption is that any parameters of subclinical disease that differ from the values for normal corneas are the result of keratoconus. However, it has not been demonstrated prospectively that all eyes in such a cohort will progress to the clinical disease state. Although true unilateral keratoconus is thought not to exist [37], this has not been proven, and it is possible that some eyes with subclinical keratoconus are not at risk of progression and that some of the abnormal parameters in this group are not the result of keratoconus. It would be valuable to conduct a prospective study in which eyes that do not develop clinical keratoconus over time are used as lower-risk examples.

#### External Validation and Generalizability to Real-world Data

To be useful, it is essential that a detection algorithm can generalize beyond the limited data set from which the model was developed and benchmarked, which requires external validation in out-of-sample data sets. The creation of a large open-source data set of keratoconus images could serve as a reference standard to develop a benchmark for external validation. We also recommend that journals adhere to the Transparent Reporting of a Multivariable Prediction Model for Individual Prognosis or Diagnosis guidelines so that all published methods are externally validated. When generalizing to external data sets the source and quality of the data should be considered. Data from a referral hospital may not represent the general population, who might be the target for screening programs, with an underrepresentation of eyes with mild disease.

#### Other Challenges

There are several other considerations, such as keratoconus progression and the translation of a detection algorithm into a medical device that can be implemented in the real world, but these issues are beyond the scope of this review. Nevertheless, these points are discussed in Multimedia Appendix 6 [37,39,93-102].

#### New Avenues of Research

On the basis of the results of this review, there is a need for further fundamental research, particularly for analysis based on the raw pixel values of corneal imaging rather than only derived parameters. Furthermore, a multimodal solution could be developed by combining these raw images with other parameters, such as biomechanical, demographic, and genetic data. Demographic data such as age, sex, ethnicity, and allergic eye disease are known risk factors for progressive keratoconus, and a family history of keratoconus is also a risk factor that should also be included in diagnostic algorithms. Environmental risk factors, including eye rubbing, have been associated with keratoconus progression, although eye rubbing is difficult to quantify. A genetic predisposition to keratoconus is supported by heritability studies in twins, linkage analysis in families, and population-level genome-wide association studies [103]. From these studies, genetic risk scores have been derived, which could be included in machine learning models for the detection of subclinical keratoconus. Ideally, a prospective study should be performed in a large cohort of young (<30 years of age) patients with subclinical keratoconus to monitor disease progression. Training should be conducted on large data sets with the explicit aim of detecting subclinical keratoconus, and the resulting model should be externally validated on a new data set. Finally, a range of machine learning techniques should be applied to the same data set along with detailed comparison statistics.

### Conclusions

We have conducted the most comprehensive review to date on machine learning algorithms for the detection of subclinical keratoconus. Early detection of keratoconus to enable treatment and prevent sight loss is a public health priority, and the use of machine learning algorithms has the potential to make the diagnostic process more efficient and widely available. We have summarized the relevant publications in terms of their input data and the choice of algorithm and identified whether studies performed appropriate validation. We have identified the challenges of obtaining accurate data sets for training machine learning algorithms and the need for a consistent, objective, and agreed definition of subclinical keratoconus. New avenues of research have been identified that combine multimodal source data with biomechanical, demographic, and genomic data. Defining disease progression and modeling progression to the point where there is sight loss are areas that may benefit from further research. We believe this up-to-date review is important to enable researchers, clinicians, and public health policymakers to understand the current state of the research and provide guidance for future health service planning.

## Appendix

Multimedia Appendix 1 Grading systems and indices.

Multimedia Appendix 2 Supplementary details for the 26 published studies that included the use of machine learning for the detection of subclinical keratoconus.

Multimedia Appendix 3 Sensitivity and specificity were plotted for the 26 published studies that included the use of machine learning for the detection of subclinical keratoconus. In the left-hand chart, the results were grouped by diagnosis criteria. A: clinically normal, topographically abnormal. B: Fellow eye of diagnosed keratoconus, clinically normal, topographically normal. C: Fellow eye of diagnosed keratoconus, clinically normal, topographically abnormal. Although there is no obvious pattern relating to diagnostic criteria, the largest outliers belong to group A, suggesting that using a fellow eye with keratoconus may lead to a better detection system. In the right-hand chart, the results are grouped according to the imaging system. No obvious pattern can be seen in the results, suggesting that the choice of imaging system is unrelated to the detection system accuracy.

Multimedia Appendix 4 Heat maps of an advanced keratoconus eye derived from Scheimpflug corneal imaging using the Pentacam device.

Multimedia Appendix 5 Results of applying the QUADAS (Quality Assessment of Diagnostic Accuracy Studies)-2 bias assessment tool including responses to tailored signaling questions.

Multimedia Appendix 6 Other challenges, such as keratoconus progression and translational considerations, have been identified for the detection of subclinical keratoconus using machine learning.

## Abbreviations

AS-OCT: anterior segment optical coherence tomography
AUC: area under the receiver operating characteristic curve
BAD-D: Belin/Ambrosio enhanced ectasia display
BFS: best-fit sphere
CNN: convolutional neural network
CXL: corneal collagen cross-linking
ESI: ectasia screening index
FFKC: forme fruste keratoconus
HOA: higher-order aberration
LASIK: laser-assisted in situ keratomileusis
NN: neural network
PRISMA: Preferred Reporting Items for Systematic Reviews and Meta-Analyses
QUADAS: Quality Assessment of Diagnostic Accuracy Studies
SVM: support vector machine
