# Supplementary material for: Machine Learning Algorithms to Detect Subclinical Keratoconus: Systematic Review
Source: JMIR Med Inform. 2021 Dec 13;9(12):e27363. doi: 10.2196/27363 (PMC8713097; doi:10.2196/27363)
Supplement: Multimedia Appendix 1 [file medinform_v9i12e27363_app1.doc]

## Grading Systems and Indices

There are a number of clinical systems to grade the severity of keratoconus, but an expert consensus report in 2015 concluded that none were clinically validated [1]. A frequently used system is Amsler-Krumeich (AK) grading, which is based on keratometry and the presence or absence of scarring [2]. However, it does not incorporate recent advances in imaging technology. The ABCD grading incorporates these imaging indices, and has a grade 0 for ‘probable absence of disease’, but does not specifically include a grade for subclinical keratoconus [3].

Placido disc-based topographic indices include Pathfinder Corneal Analysis, keratoconus percentage index (KISA%), KPI and KCI indices, and I-S value [4]. These indices are based on anterior elevation and pachymetry and thus may miss earlier signs of keratoconus such as posterior elevation change and horizontal displacement the zone of thinning [4].

The tomography based Belin/Ambrósio Enhanced Ectasia Display (BAD) is an inbuilt function in the Pentacam (OCULUS GmbH, Wetzlar Germany) designed to generate a comprehensive display for keratoconus and ectasia screening through the analysis of multiple variables [4]. Regression analysis using a database of parameters from normal individuals and patients with keratoconus generates a numeric ‘D’ value (BAD_D), which indicates how the examined cornea varies from ‘normal’. However, it was not specifically designed to identify subclinical keratoconus, and has 81.1% sensitivity and 73.2% specificity for distinguishing suspect-keratoconus from controls [5].

Other grading systems based on the Pentacam include the topographic keratoconus classification (TKC) [6]. When considering other imaging systems, the Ocular Response Analyzer keratoconus match index (KMI) is based on a composite value of seven parameters derived from the reflected waveform generated as the cornea is distorted by a puff of air [7].

1. Gomes JAP, Tan D, Rapuano CJ, Belin MW, Ambrósio R Jr, Guell JL, Malecaze F, Nishida K, Sangwan VS, Group of Panelists for the Global Delphi Panel of Keratoconus and Ectatic Diseases. Global consensus on keratoconus and ectatic diseases. Cornea 2015 Apr;34(4):359–369. PMID:25738235

2. Amsler M. Kératocône classique et kératocône fruste; arguments unitaires. Ophthalmologica 1946;111(2–3):96–101.

3. Belin MW, Duncan JK. Keratoconus: The ABCD Grading System. Klin Monbl Augenheilkd 2016 Jun;233(6):701–707. PMID:26789119

4. IndependentPopulationValidationoftheBelinAmbrosioEnhancedEctasiaDisplayImplicationsforKeratoconusStudiesandScreening.pdf [Internet]. [doi: 10.5005/jp-journals-10025-1069]

5. Hashemi H, Beiranvand A, Yekta A, Maleki A, Yazdani N, Khabazkhoob M. Pentacam top indices for diagnosing subclinical and definite keratoconus. Journal of Current Ophthalmology 2016 Mar 1;28(1):21–26.

6. OCULUS. Pentacam® Core Functions [Internet]. Pentacam. 2020 [cited 2020]. Available from: https://www.pentacam.com/int/opticianoptometrist-without-pentacamr/models/pentacamr/core-functions.html

7. Reichert Technologies. Ocular Response Analyzer, User’s Guide. 2012.
