# Supplementary figures and images for: Machine Learning Algorithms to Detect Subclinical Keratoconus: Systematic Review
Source: JMIR Med Inform. 2021 Dec 13;9(12):e27363. doi: 10.2196/27363 (PMC8713097; doi:10.2196/27363)

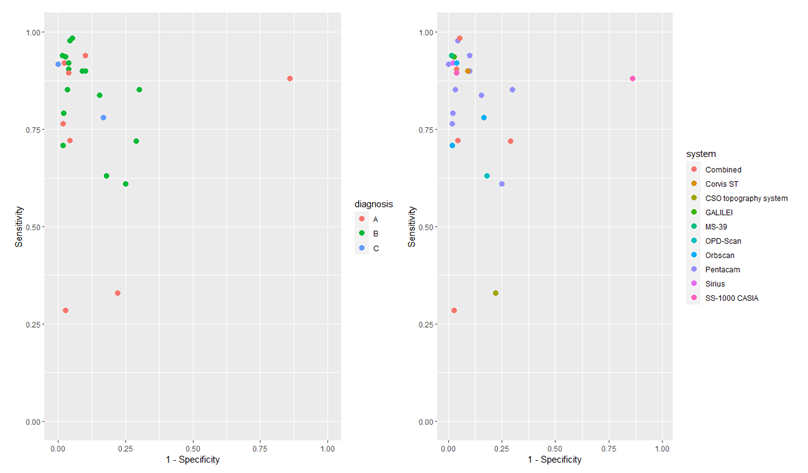

Supplement: Multimedia Appendix 3 [file medinform_v9i12e27363_app3.png]

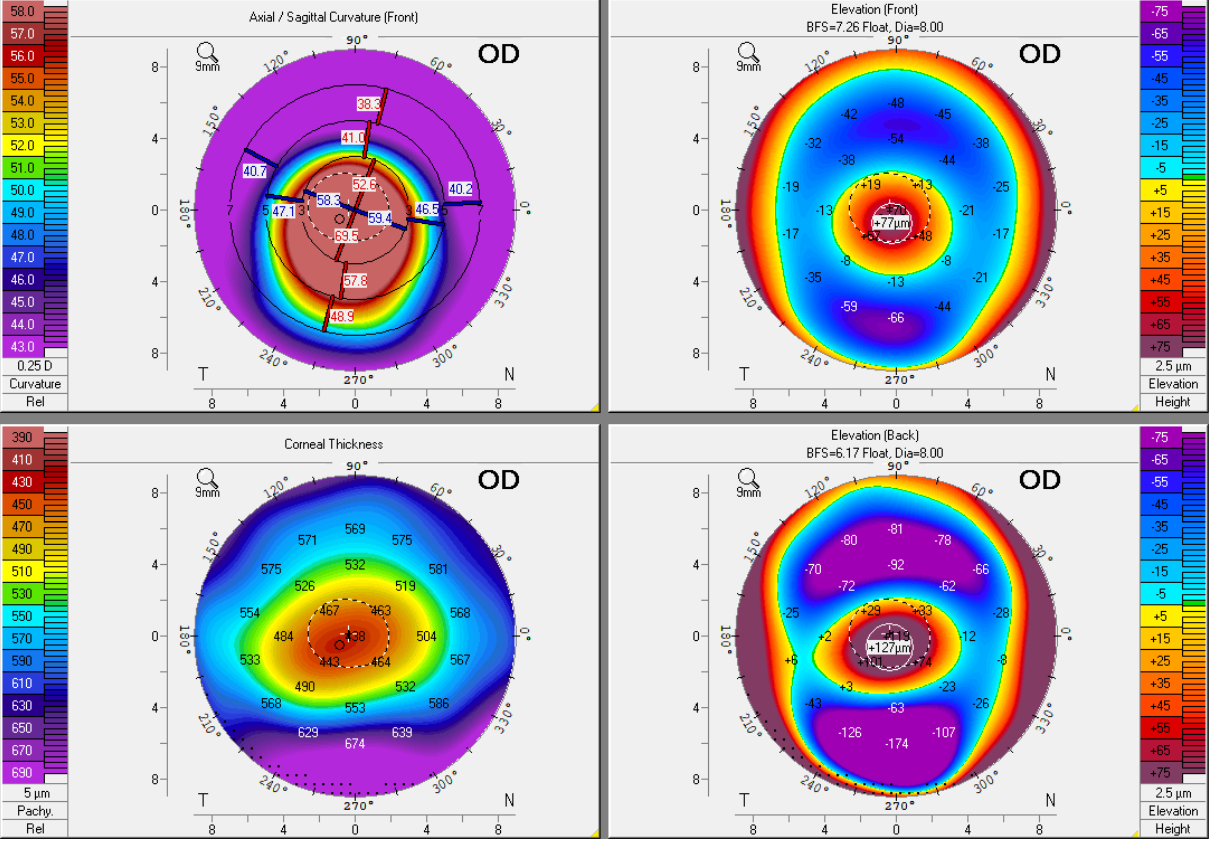

Supplement: Multimedia Appendix 4 [file medinform_v9i12e27363_app4.png]
