# Supplementary material for: Machine Learning Algorithms to Detect Subclinical Keratoconus: Systematic Review
Source: JMIR Med Inform. 2021 Dec 13;9(12):e27363. doi: 10.2196/27363 (PMC8713097; doi:10.2196/27363)
Supplement: Multimedia Appendix 6 [file medinform_v9i12e27363_app6.docx]

#### Progression

Keratoconus is a progressive disease and, as such, detecting signs of progression is important for early diagnosis and it is well known that younger patients and paediatric cases tend to be faster progressors [1]. According to the Global Consensus of Keratoconus and Ectatic Diseases, progression is defined by a consistent change in at least two of the following parameters: steepening of anterior corneal surface, steepening of posterior corneal surface, thinning and/or an increase in the rate of corneal thickness change [2]. Increasing K_max_ is the most commonly used parameter for defining steepening of the corneal surface [3]. However some authors believe that K_max_ is an unreliable parameter for detecting progression because keratometry has been shown to have poor repeatability in established keratoconus patients [4–7]. Other measures include visual acuity and pachymetry, but they have been found to be poor indicators of keratoconus progression [8]. In addition, monitoring the anterior cornea surface with topometric indices such as ISV and IHD has been put forward as the best performing indicators of progression [9].

Similar to subclinical keratoconus detection, using a combination of parameters might lead to promising results. Pinero et al. suggested that corneal astigmatism changes brought about by keratoconus progression must be considered in more than one dimension, and hence lends itself to vector analysis by defining both magnitude and axis. Using three vectors, Target-Indices Astigmatism (TIA), Surgically Induced Astigmatism (SIA) and Difference Vector (DV), as input to a regression model, the authors detected worsening of keratoconus with a sensitivity of 63.2% and specificity of 53.9% [10].

Very little has been published concerning the application of machine learning methods to detect keratoconus progression. When considering glaucoma progression, machine learning algorithms have been used to detect visual field progression earlier than other methods [11] and Markov random fields have been employed to exploit the spatial voxel dependency from 3D spectral domain OCT (SD-OCT) images [12]. In other fields of medicine, recurrent neural networks such as Long Short-Term Memory models are capable of diagnosing disease given a time series of data [13]. This could be applied to keratoconus progression where the inputs to these networks might either be a temporal sequence of parameters (e.g. keratometry, pachymetry etc.) or sequential images. These relatively novel algorithms have also been applied to medical videos such as ultrasound but not yet to ophthalmology time series [14].

#### Importance of Predicting and Implications for Screening

The ability to identify individuals who are at high risk of developing keratoconus would be a significant advance in clinical care with public health implications given the existence of a cost-effective low risk treatment. In diabetic retinopathy screening, which is well established, health economic analysis has demonstrated that a semi-automated model is more cost-effective compared to human graders alone, but as yet no health economic studies on a purely automated system has been published [15].

If reliable machine learning algorithms could be developed using available imaging techniques, there would be a role for screening the population at risk (e.g., ages 10 to 30 years, particularly those with an atopic history and higher risk ethnicities). Artificial intelligence assistive technologies have started to be deployed elsewhere in ophthalmology, such as the Food and Drug Administration (FDA) approved diabetic retinopathy screening deep-learning system [16] and the deep learning system for identification of retinopathy of prematurity that has achieved FDA breakthrough status [17].

#### Underdiagnosis and Overdiagnosis

The purpose of a machine learning system to detect subclinical keratoconus is to identify disease at a stage when treatment can prevent sight loss. Minimising the rate of underdiagnosis (false negatives) is therefore paramount, but the potential for harm from overdiagnosis (false positives) should not be overlooked, and the expense from over-investigation and treatment is a widespread problem in modern healthcare [18]. It is also important to quantify the patient benefit associated with the adoption of any new investigation, which cannot be ignored. For example, twenty years after FDA approval for computer aided detection (CAD) systems for mammography screening, a follow up study concluded that the CAD system can miss cancers while insurers pay more for CAD than for the prior established methods with no benefit to patients [19]. Whilst large randomized clinical trials to address these questions are not always justified or feasible for some diseases [20], in the case of subclinical keratoconus, it is eminently possible.

#### Roadmap to software as a medical device

Before an algorithm can be developed into software as a medical device (SaMD), it needs to be developed according to regulatory processes such as ISO 13485, which require detailed documentation, risk assessment and auditing of the software development process. Furthermore, to receive approval from regulatory medical agencies, such as FDA in the US, EMA in the EU or MHRA in the UK, safety and efficacy needs to be thoroughly assessed [21]. The first phase can be achieved in a randomized case-control trial in a controlled clinical environment to assess safety (the software does not lead to dangerous decisions for the patient) and efficacy (clinical pathways that include the software lead to improved outcomes over current practice). Further to this, health technology assessment studies can be conducted to assess the generalisability and utility of the software in a range of clinical environments (e.g. specialist hospital or optometry practices), how it influences decision-making and enhances the clinical pathway toward monitoring of disease or treatment (e.g. CXL), and whether it is economically viable. When sufficient evidence of safety and efficacy has been generated and if the software fulfils the conformity assessment, it may obtain SaMD accreditation and CE marking. In the UK, following expert consultation and review, approval by NICE can lead to widespread adoption within the national healthcare setting [16,20]. Currently, to the best of our knowledge, no detection algorithm for subclinical keratoconus has been taken beyond a proof of concept stage demonstrating diagnostic efficacy [21,22].

1. Ahn SJ, Kim MK, Wee WR. Topographic progression of keratoconus in the Korean population. Korean J Ophthalmol 2013 Jun;27(3):162–166. PMID:23730107

2. Gomes JAP, Tan D, Rapuano CJ, Belin MW, Ambrósio R Jr, Guell JL, Malecaze F, Nishida K, Sangwan VS, Group of Panelists for the Global Delphi Panel of Keratoconus and Ectatic Diseases. Global consensus on keratoconus and ectatic diseases. Cornea 2015 Apr;34(4):359–369. PMID:25738235

3. Duncan JK, Belin MW, Borgstrom M. Assessing progression of keratoconus: novel tomographic determinants. Eye Vis (Lond) 2016 Mar 11;3:6. PMID:26973847

4. Flynn TH, Sharma DP, Bunce C, Wilkins MR. Differential precision of corneal Pentacam HR measurements in early and advanced keratoconus. Br J Ophthalmol 2016 Sep;100(9):1183–1187. PMID:26659714

5. Meyer JJ, Gokul A, Vellara HR, Prime Z, McGhee CNJ. Repeatability and Agreement of Orbscan II, Pentacam HR, and Galilei Tomography Systems in Corneas With Keratoconus. Am J Ophthalmol 2017 Mar;175:122–128. PMID:27993593

6. Guilbert E, Saad A, Elluard M, Grise-Dulac A, Rouger H, Gatinel D. Repeatability of Keratometry Measurements Obtained With Three Topographers in Keratoconic and Normal Corneas. J Refract Surg 2016 Mar;32(3):187–192. PMID:27027626

7. Hashemi H, Yekta A, Khabazkhoob M. Effect of keratoconus grades on repeatability of keratometry readings: Comparison of 5 devices. J Cataract Refract Surg 2015 May;41(5):1065–1072. PMID:26049838

8. Kanellopoulos AJ, Moustou V, Asimellis G. Evaluation of Visual Acuity, Pachymetry and Anterior- Surface Irregularity in Keratoconus and Crosslinking Intervention Follow-up in 737 Cases. unknown; 2013 Dec 1;2(3):95–103.

9. Kanellopoulos AJ, Asimellis G. Revisiting keratoconus diagnosis and progression classification based on evaluation of corneal asymmetry indices, derived from Scheimpflug imaging in keratoconic and suspect cases. Clin Ophthalmol 2013 Jul 26;7:1539–1548. PMID:23935360

10. Piñero DP, Alio JL, Tomás J, Maldonado MJ, Teus MA, Barraquer RI. Vector analysis of evolutive corneal astigmatic changes in keratoconus. Invest Ophthalmol Vis Sci 2011 Jun 8;52(7):4054–4062. PMID:21372010

11. Yousefi S, Kiwaki T, Zheng Y, Sugiura H, Asaoka R, Murata H, Lemij H, Yamanishi K. Detection of Longitudinal Visual Field Progression in Glaucoma Using Machine Learning. Am J Ophthalmol 2018 Sep;193:71–79. PMID:29920226

12. Belghith A, Bowd C, Medeiros FA, Balasubramanian M, Weinreb RN, Zangwill LM. Glaucoma progression detection using nonlocal Markov random field prior. J Med Imaging (Bellingham) 2014 Oct;1(3):034504. PMID:26158069

13. Lipton ZC, Kale DC, Elkan C, Wetzel R. Learning to Diagnose with LSTM Recurrent Neural Networks [Internet]. arXiv [csLG]. 2015. Available from: http://arxiv.org/abs/1511.03677

14. Liu S, Wang Y, Yang X, Lei B, Liu L, Li SX, Ni D, Wang T. Deep Learning in Medical Ultrasound Analysis: A Review. Proc Est Acad Sci Eng 2019 Apr 1;5(2):261–275.

15. Xie Y, Gunasekeran DV, Balaskas K, Keane PA, Sim DA, Bachmann LM, Macrae C, Ting DSW. Health economic and safety considerations for artificial intelligence applications in diabetic retinopathy screening. Transl Vis Sci Technol Association for Research in Vision and Ophthalmology (ARVO); 2020 Apr;9(2):22. PMID:32818083

16. Abràmoff MD, Lavin PT, Birch M, Shah N, Folk JC. Pivotal trial of an autonomous AI-based diagnostic system for detection of diabetic retinopathy in primary care offices. NPJ Digit Med 2018 Aug 28;1:39. PMID:31304320

17. Brown JM, Campbell JP, Beers A, Chang K, Ostmo S, Chan RVP, Dy J, Erdogmus D, Ioannidis S, Kalpathy-Cramer J, Chiang MF, Imaging and Informatics in Retinopathy of Prematurity (i-ROP) Research Consortium. Automated Diagnosis of Plus Disease in Retinopathy of Prematurity Using Deep Convolutional Neural Networks. JAMA Ophthalmol 2018 Jul 1;136(7):803–810. PMID:29801159

18. Brodersen J, Schwartz LM, Heneghan C, O’Sullivan JW, Aronson JK, Woloshin S. Overdiagnosis: what it is and what it isn’t. BMJ Evid Based Med 2018 Feb;23(1):1–3. PMID:29367314

19. Lehman CD, Wellman RD, Buist DSM, Kerlikowske K, Tosteson ANA, Miglioretti DL, Breast Cancer Surveillance Consortium. Diagnostic accuracy of digital screening mammography with and without computer-aided detection. JAMA Intern Med American Medical Association (AMA); 2015 Nov;175(11):1828–1837. PMID:26414882

20. Keane PA, Topol EJ. With an eye to AI and autonomous diagnosis. NPJ Digit Med 2018 Aug 28;1:40. PMID:31304321

21. Larson DB, Harvey H, Rubin DL, Irani N, Tse JR, Langlotz CP. Regulatory Frameworks for Development and Evaluation of Artificial Intelligence-Based Diagnostic Imaging Algorithms: Summary and Recommendations. J Am Coll Radiol [Internet] 2020 Oct 20; PMID:33096088

22. Fryback DG, Thornbury JR. The efficacy of diagnostic imaging. Med Decis Making 1991 Apr;11(2):88–94. PMID:1907710
